# First Detection of *Cytauxzoon* spp. DNA in Questing *Ixodes ricinus* Ticks

**DOI:** 10.3390/microorganisms13092188

**Published:** 2025-09-19

**Authors:** Marina L. Meli, Theres Meili, Benita Pineroli, Eva Boenzli, Ramon M. Eichenberger, Barbara Willi, Regina Hofmann-Lehmann

**Affiliations:** 1 Clinical Laboratory, Department of Clinical Diagnostics and Services, and Center for Clinical Studies, Vetsuisse Faculty, University of Zurich, Winterthurerstrasse 260, 8057 Zurich, Switzerland; tmeili@vetclinics.uzh.ch (T.M.); bpineroli@vetclinics.uzh.ch (B.P.); eva.boenzli@gmx.ch (E.B.); regina.hofmann-lehmann@uzh.ch (R.H.-L.); 2 Institute of Parasitology, Vetsuisse-Faculty, University of Zurich, Winterthurerstrasse 266a, 8057 Zurich, Switzerland; 3 Medical Micro- and Molecular Biology, Institute of Chemistry and Biotechnology, Zurich University of Applied Sciences (ZHAW), Einsiedlerstrasse 31, 8820 Wädenswil, Switzerland; 4 Clinic for Small Animal Internal Medicine, Vetsuisse Faculty, University of Zurich, Winterthurerstrasse 260, 8057 Zurich, Switzerland; bwilli@vetclinics.uzh.ch

**Keywords:** vector-borne disease, infectious anemia, hemoparasite, apicomplexa, felids, *Cytauxzoon* spp., questing ticks, *Ixodes ricinus*, Switzerland

## Abstract

Feline cytauxzoonosis is an emerging tick-borne disease in Europe. While infections have been reported in different European countries, the tick vector remains unknown. This study investigated 665 ticks collected in 2019 (n = 160), 2022 (n = 7), and 2024 (n = 498) in a *Cytauxzoon* spp. hotspot region in central Switzerland (62 ticks from cats; 603 ticks from vegetation). Ticks were morphologically characterized, pooled by origin and life-stage, and screened for *Cytauxzoon* spp. 18S rRNA by qPCR and conventional PCR, and positive samples confirmed by sequencing. All ticks belonged to *Ixodes ricinus* (50 males, 83 females, 532 nymphs). Four tick pools from 2019 tested *Cytauxzoon* spp. positive: one pool of 3 non-engorged male ticks from two cats and three pools of 5–6 nymphs each from vegetation. All ticks collected in 2022 and 2024 tested negative. Amplification of the almost full-length (1535 bp, one pool) or partial (140–219 bp, three pools) 18S rRNA gene revealed a sequence identity of 98.6–100% with *Cytauxzoon* spp. previously detected in cats from this area. The detection of *Cytauxzoon* spp. in questing *I. ricinus* nymphs suggests a potential role of this tick species in the parasites’ transmission cycle in Central Europe and raises the possibility of transstadial or potentially transovarial transmission. Mitochondrial gene sequencing was unsuccessful, but the detected *Cytauxzoon* spp. likely represent *Cytauxzoon europaeus*. Discrepancies between qPCR and conventional PCR results point to possible amplification of tick endosymbionts, highlighting the importance of confirmatory sequencing, particularly when testing tick-derived DNA. Thus, the 18S rRNA qPCR assay used appears suboptimal for screening tick samples, as its specificity in this matrix was limited. In conclusion, this is the first report of *Cytauxzoon* spp. in questing *I. ricinus* ticks in Europe. Our findings underscore the need for further research to confirm vector competence and clarify transmission dynamics.

## 1. Introduction

*Cytauxzoon* species (Apicomplexa, Piroplasmida, Theileriidae) [1] are vector-borne apicomplexan hemoparasites that infect both wild and domestic felids globally. Seven species within the genus *Cytauxzoon* have been identified. Among them, *Cytauxzoon felis* is known to cause severe and often fatal disease in domestic cats and has been well characterized in domestic and wild felids in the United States [2]. The bobcat (*Lynx rufus*)—which typically experiences asymptomatic infections—is considered the natural reservoir of *C. felis* in the United States, but other wild felid species (mountain lions, cougars, ocelots, margays, and jaguars), as well as subclinically infected domestic cats, may also serve as reservoirs [3,4,5,6,7,8,9,10]. In recent years, organisms closely related to *C. felis* have been reported in domestic cats in China, India, and Iran, and in domestic and wild felids in Brazil [11,12,13,14]. Collectively, these *Cytauxzoon* spp. isolates from Asia and South America are phylogenetically distinct, belonging to different clades, from those described in Eurasia. The latter comprise *Cytauxzoon manul* isolated from a Pallas’s cat (*Otocolobus manul*) from Mongolia [15], and *Cytauxzoon* spp. isolated from domestic and wild cats in different regions in Europe [16,17,18,19,20,21,22,23,24,25,26,27]. Most recently, two new species of the genus *Cytauxzoon brasiliensis* in wild felids in Brazil and Argentina, and *Cytauxzoon* sp. “Kozhikode” in domestic cats in India [11,12,28] have been described.

The first reports of cytauxzoonosis in Europe date back to 2003, with the detection of *Cytauxzoon* spp. in the Iberian lynx (*Lynx pardinus*) [29,30,31] and in domestic cats in Spain [32]. Subsequently, retrospective analyses revealed that *Cytauxzoon* spp. infections were already present in French wildcats as early as 1995, with a reported prevalence of 29%, and in a Swiss domestic cat in 2003 [25]. Since then, *Cytauxzoon* spp. have been identified in stray and domestic cats, as well as in free-ranging and captive wild felids, across various European countries, including Italy, Portugal, Spain, France, Switzerland, Germany, Hungary, and the European part of Russia [16,17,18,19,20,21,22,23,24,25,26,27]. In Eurasia, reservoir hosts include wild felids such as the Iberian lynx, the Eurasian lynx (*Lynx lynx*), and the European wildcat (*Felis silvestris*), as well as chronically infected domestic cats exhibiting prolonged parasitemia without clinical signs [20,33].

Phylogenetic analyses based on mitochondrial gene sequences (cytochrome b and cytochrome oxidase subunit I) have revealed the presence of three distinct *Cytauxzoon* species circulating in wild felids in Europe: *Cytauxzoon europaeus* (EU1), *Cytauxzoon otrantorum* (EU2), and *Cytauxzoon banethi* (EU3) [34]. Among these, *C. europaeus* seems to be most prevalent and is the only species detected so far in domestic cats in Europe. *Cytauxzoon europaeus* infection has been reported in wild and domestic felids in Hungary, France, Switzerland, Germany, Romania, the Czech Republic, Bosnia and Herzegovina, and Italy [25,35,36,37,38,39].

European *Cytauxzoon* spp. are generally regarded as less pathogenic than *C. felis* [21]. However, clinical disease and fatal cases have been documented in domestic cats [16,18,20,21,33]. Infections with *Cytauxzoon* spp. are characterized by an asexual replication in the host’s mononuclear phagocytic cells. Massive numbers of schizont-laden mononuclear cells that obstruct the vascular lumen of different organs are responsible for the severe clinical signs observed in domestic cats infected with *C. felis* [40]. Notably, this schizogonous stage has not been observed so far in domestic or wild felids infected with European *Cytauxzoon* spp. [16,20,33], suggesting that the schizogonous phase in European *Cytauxzoon* spp. is probably more limited. This has also been reported for *C. felis* infections in bobcats, which often go asymptomatic [41].

In the United States, *C. felis* is transmitted by ticks (Arthropoda, Ixodida, Ixodidae), with *Amblyomma americanum* and *Dermacentor variabilis* identified as competent vectors. Transstadial transmission has been demonstrated in these species, and ticks can transmit the parasite from both clinically ill and subclinically infected cats to susceptible hosts [42,43,44,45], while infected wild felids may act as reservoirs. In contrast, the tick vector responsible for the transmission of *Cytauxzoon* spp. in Europe remains unidentified. However, tick species such as *Dermacentor* spp., *Ixodes* spp., and *Rhipicephalus* spp.—all of which are present in Europe—are considered potential vectors [46,47].

The aim of this study was to investigate the presence of *Cytauxzoon* spp. in ticks collected in a hotspot region of cytauxzoonosis in domestic cats in Switzerland in 2019 and in the same area during subsequent seasons. The ticks were morphologically characterized, pooled according to origin and life stage, and the pools were investigated for the presence of *Cytauxzoon* spp. 18S rRNA by qPCR. In positive pools, 18S rRNA gene sequencing and sequencing of the mitochondrial genes (CytB and COI) were attempted.

## 2. Materials and Methods

### 2.1. Sample Collection and Characteristics

**Figure 1:**
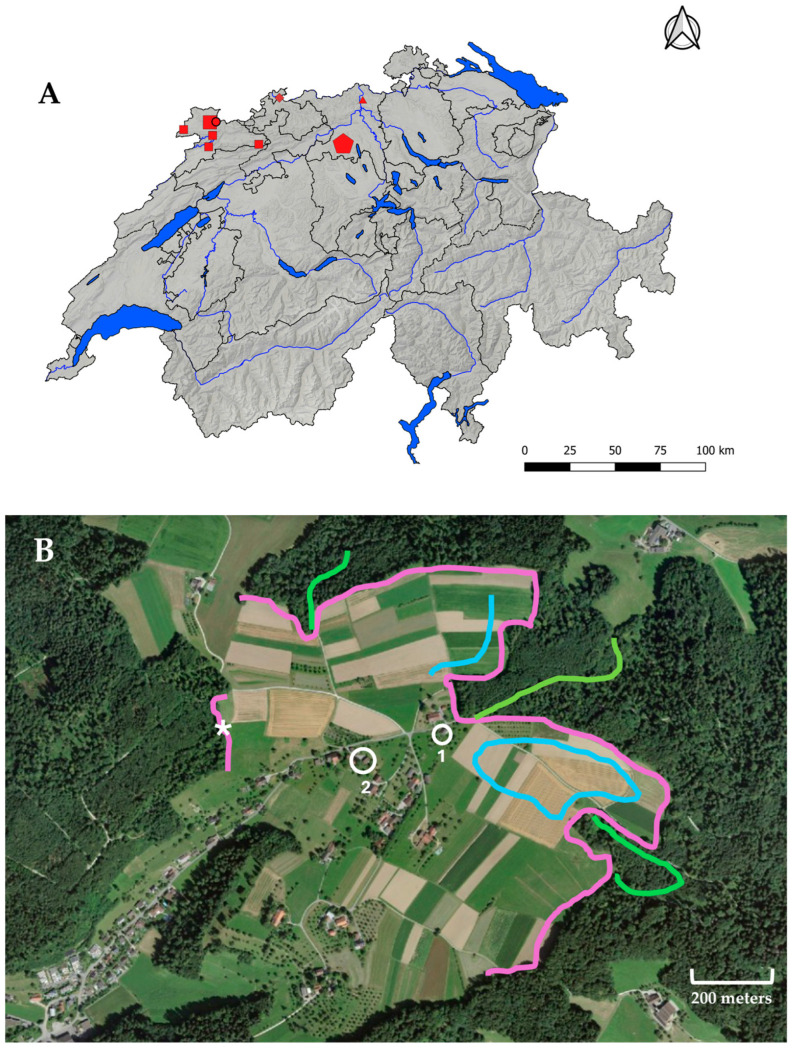
Map of Switzerland (**A**) showing the geographical distribution of the positive domestic and stray cat samples [25] and of the hotspot region (**B**) where the ticks were collected. (**A**) The geographic origin of the *Cytauxzoon* spp. positive cats from the previous study are displayed. Pentagon: region of the hotspot where the ticks were collected; circle: positive domestic cat of the Swiss-wide study 2013–2016; rhomb: positive anemic cat from the study 2019–2021; squares: stray domestic cats 2014; triangle: positive domestic cat from 2003. The size of the symbols indicates the number of *Cytauxzoon* spp. PCR-positive samples per location. (**B**) Detailed satellite map (https://map.geo.admin.ch/; accessed on 17 July 2025) of the region of the hotspot with the two neighboring households (white circles 1 and 2 in CH-5726 Unterkulm, Google Plus coordinates household 2: 47.317590, 8.128228) and the regions where the ticks were collected. Pink: forest edges; green: forest paths; blue: meadow. White asterisk: region where the 3 positive questing tick pools (Nos.: 13, 17, 19) were collected.

In February and March 2019, a total of 160 ticks were collected in a rural area close to Unterkulm, a village located in central Switzerland. A total of 62 ticks were directly collected from 7 cats by their owner from a household, in which we recently documented *C. europeaus* infections in 3 out of 10 cats (household 2 [25]). The use of leftover material in scientific projects has been approved by the Ethics Committee of the Faculty of Medicine, University of Zurich (MeF-Ethik-2024-14). Animal owners gave their consent for the use of data and residual sample material of their animals. Furthermore, 98 questing ticks were collected from the vegetation at the edge of a forest located around 350 m away from this household. The questing ticks were collected by dragging a 1 m^2^ white cotton cloth over the vegetation (dragging method [48]). An additional 505 questing ticks were collected during subsequent years from the same area (7 in September 2022, and 498 in May 2024): 423 from the edge of the forest, 33 from the meadow near the forest, and 49 from forest paths. A map of the so far detected *Cytauxzoon* spp. positive domestic and stray cats in Switzerland (adapted from [25]) and a detailed map of the region where the ticks were collected is displayed in Figure 1A and Figure 1B, respectively. Ticks were transferred to 1.5 mL Eppendorf screw tubes pre-filled with 70% ethanol and stored at room temperature until further processing, as previously described [49]. All ticks were morphologically characterized by one of the authors (R.M.E) according to published methods [50,51,52].

### 2.2. Nucleic Acid Extraction

DNA from engorged ticks and tick pools was extracted using the QIAamp^®^ DNA Mini Kit (Qiagen, Hombrechtikon, Switzerland) according to the manufacturer’s instructions, with some modifications as previously described [53]. Briefly, tick pools were pre-processed as follows: all ticks were first air-dried and then sequentially washed in 10% bleach, tap water, and distilled water [49]. Air-dried ticks were then minced with a sterile scalpel on parafilm tape and transferred into a sterile 2 mL round-bottomed tube. A 5 mm steel bead (Retsch, Haan, Germany) and 200 μL ATL buffer were added to the minced material. The ticks were disrupted using a tissue homogenizer: either a Mixer-Mill 300 (Retsch) or a Precellys^®^ 24 tissue homogenizer (Bertin Technologies SAS, Montigny-le-Bretonneux, France) set to 30 Hz for 1 min. After a short centrifugation step to remove possible material from the tube lid, the steel bead was removed, and 20 μL proteinase K (Qiagen) was added, and the samples were digested overnight at 56 °C. Buffer AL (400 μL) was added to the completely lysed samples and mixed thoroughly by vortexing for 15 s and incubated for 10 min at 70 °C. After a short centrifugation step to remove drops from the lid, 400 μL 96% ethanol was added and mixed, and centrifuged again before loading the mixture onto the QIAamp Mini spin column (Qiagen). After the washing procedures, DNA was eluted from the column using 100 μL buffer AE and 5 min incubation at room temperature. DNA was stored at −80 °C until further processing. At each extraction batch, a negative extraction control, consisting of 200 μL of Hank’s Balanced Salt Solution (1x HBSS, Gibco, Thermofisher Scientific, Basel, Switzerland), was run in parallel.

### 2.3. Diagnostic Assays, Amplification of the 18S rRNA, CytB and COI Genes, and Sequencing

Quality and quantity of the DNA samples extracted from ticks were tested with a commercial 18S rRNA real-time TaqMan qPCR assay (Thermofisher Scientific) as described previously [54]. Only samples with cycle threshold (Ct) values < 30 were further analyzed and screened for *Cytauxzoon* spp. using a real-time TaqMan qPCR assay that amplifies 69 bp of the 18S rRNA gene [21,25,30]. Assays were run on an ABI 7500 Fast Real-Time PCR system (Applied Biosystems, Rotkreuz, Switzerland). Positive and negative PCR controls were run with each PCR assay and consisted of DNA from a *Cytauxzoon* spp. PCR-positive Iberian lynx (confirmed by sequencing) and nuclease-free water, respectively. All samples with threshold cycle (Ct) values < 35 were subjected to confirmation by a conventional PCR that amplifies 221 bp of the 18S rRNA gene of *Cytauxzoon* spp. [21,30].

Samples that were PCR positive in the 18S rRNA confirmatory conventional PCR were sequenced (221 bp) and underwent amplification and sequencing of the almost complete 18S rRNA gene (1637 bp) as previously described [21], cytochrome b (CytB) and cytochrome oxidase subunit I (COI) mitochondrial genes of *Cytauxzoon* spp., either directly or after a pre-amplification step using the Prelude™ PreAmp Master Mix (TaKaRa Bio, Kusatsu, Japan), as previously described [25]. The PCR assays and the primers used are summarized in Table 1. The PCR products were separated on a 2% agarose gel, and bands of appropriate size were sequenced at a commercial laboratory (Microsynth AG, Balgach, Switzerland) using the amplification primers. Sequences were edited and assembled using Geneious Prime^®^ 2020.2.5 software (https://www.geneious.com, accessed on 17 July 2025; Biomatters Limited, Auckland, New Zealand) [55]. Sequence identification was conducted by comparing the obtained sequences to existing sequences through the BLASTn search program (http://www.ncbi.nlm.nih.gov/blast/Blast.cgi, accessed on 17 July 2025).

Nucleotide sequences obtained in this study, with the exception of the sequence from pool 19 (too short for submission, <200 bp), have been submitted to GenBank under accession numbers PV944017 (tick pool 7), PV944018 (tick pool 13), and PV944019 (tick pool 17).

## 3. Results

### 3.1. Ticks Collected in the Hotspot Region in 2019

A total of 160 ticks collected in 2019 were analyzed. All ticks belonged to the species *I. ricinus* and comprised 14 males, 49 females and 97 nymphs. They were allocated to 42 pools according to origin, sex, and developmental stage (Table 2). Sixty-two ticks were collected from domestic cats residing in one of the households in which *C. europaeus* infection was documented in several cats [25]. An additional 98 ticks (2 females and 96 nymphs) were collected from vegetation at the forest edge, approximately 350 m northwest of the household.

All 42 tick pools tested positive for *Cytauxzoon* spp. using qPCR screening and were subsequently analyzed by conventional PCR targeting a 221 bp fragment of the 18S rRNA gene. Of these, four pools yielded weakly positive amplicons. Sequencing confirmed the presence of *Cytauxzoon* spp. in these four pools, with three yielding partial sequences of 140–219 bp and one yielding an almost full-length sequence of 1535 bp of the 18S rRNA gene (Table 2). The four *Cytauxzoon*-positive pools included one pool (No. 7) containing 4 non-engorged male *I. ricinus* ticks collected from two domestic cats of unknown *Cytauxzoon* spp. infection status, and three pools (Nos. 13, 17, and 19) containing 5–6 *I. ricinus nymphs* each collected from vegetation.

BLAST analysis of the 1535 bp sequence of pool No. 7 revealed 100% identity with *Cytauxzoon* spp. sequences previously obtained from two domestic cats in the neighboring household, as well as from two French wildcats sampled in 1995 [25]. Additionally, this sequence was identical to those of *Cytauxzoon* spp. isolated from two Italian domestic cats collected in 2016/2017 (unpublished dataset, GenBank accession numbers OM004051 and OM004053).

The partial sequences from tick pools No. 13, 17, and 19 showed 98.6%, 99.5%, and 99.3% identity, respectively, to *Cytauxzoon* spp. sequences—including *Cytauxzoon europaeus*—previously detected in European wild felids [25,34,38,39] and Swiss domestic cats [21,25]. Pairwise comparison of the partial sequences obtained in this study showed from 97.9% to 99.8% sequence identity.

All attempts to amplify and sequence the mitochondrial genes (CytB and COI) from the positive pools were unsuccessful.

### 3.2. Ticks Collected in 2022 and 2024

In 2022 and 2024—approximately three and a half and five years after the first collection in the endemic region [25]—an additional 505 ticks were collected from the same area and analyzed for the presence of *Cytauxzoon* spp.

All 505 ticks belonged to the species *I. ricinus* and comprised 36 males, 34 females, and 435 nymphs. They were allocated to 143 pools according to origin, sex, and developmental stage (Table 3). All 143 tick pools tested positive for *Cytauxzoon* spp. using qPCR screening and were subsequently analyzed by conventional PCR targeting a 221 bp fragment of the 18S rRNA gene. However, none of the individual ticks or pooled samples yielded a positive result in the conventional PCR assay (Table 3).

## 4. Discussion

This study reports, for the first time, the detection of *Cytauxzoon* spp. in questing *I. ricinus* ticks. To date, the tick vector for European *Cytauxzoon* spp. is unknown. *Cytauxzoon* spp. were detected by conventional 18S rRNA PCR in ticks collected from cats living in households with *C. europaeus*-infected cats, but also in questing ticks collected in an area located around 350 m (~380 yards) away from this household. Subsequent sequencing of the amplified fragments of the 18S rRNA gene confirmed the taxonomic assignment to the *Cytauxzoon* genus. Despite DNA pre-amplification, species-level identification could not be achieved, most likely due to the extremely low parasitic load in the tick samples, below the detection limit for the mitochondrial gene-specific primers, or possibly due to partial DNA degradation. Given that only *C. europaeus* has been reported in domestic and wild felids in Switzerland [25], and that the positive ticks were collected concurrently with the occurrence of *C. europaeus* infections in several cats of the household, the detected organisms in the ticks likely represent *C. europaeus*. Nonetheless, we were unable to amplify and sequence the mitochondrial genes COI and CytB of *Cytauxzoon* spp. from the positive tick pools, which would be important for confirming the assignment of the detected species as *C. europeaus*. Therefore, a second attempt was undertaken in autumn 2022 and spring 2024 to collect more questing ticks from the same area, where cytauxzoonosis was considered to be endemic [25]. However, no further *Cytauxzoon*-positive ticks were identified at that time. Given that infected cats typically remain PCR-positive for *Cytauxzoon* spp. for years, even after antibiotic treatment [21], it was expected that these chronically infected animals with prolonged parasitemia would contribute to a sustained parasitic burden within the local tick population. However, possibly increased implementation of tick prophylaxis measures—potentially prompted by the earlier *Cytauxzoonosis* infection—may have significantly reduced tick exposure in domestic animals, leading to a subsequent decrease in tick infestation rates and, consequently, a lower prevalence of *Cytauxzoon* spp. in the tick population.

*Cytauxzoon felis* is transmitted by *D. variabilis* and *A. americanum* ticks [42,43], neither of which occurs in Central Europe [47]. *Ixodes ricinus* is by far the most common tick species in Switzerland and is present in most parts of the country [56], but also highly prevalent in other central European countries [47]. *Ixodes ricinus* ticks are known vectors of many pathogens of veterinary relevance, among them other piroplasms like *Babesia* (i.e., *Babesia divergens*, *Babesia microti*, and *Babesia venatorum* [57,58,59]), as well as *Theileria* spp. [60]. *Babesia* spp. are phylogenetically related to *Cytauxzoon* spp., but they differ in their life cycle compared to *C. felis*, since *Babesia* spp. do not undergo a schizogenous stage. However, schizogony has not yet been documented in domestic and wild felids infected with European *Cytauxzoon* spp. [25]. Besides *I. ricinus*, *Dermacentor marginatus*, *Dermacentor reticulatus*, *Haemaphysalis punctata*, *Rhipicephalus sanguineus*, and other species of the genus *Ixodes* spp. (*Ixodes hexagonus*) have also been described in Switzerland [61,62,63], and *H. punctata* has been shown to transmit *Babesia major* [64]. In this study, 603 questing *I. ricinus* ticks were collected, predominantly nymphs (88%), with only a few adults. Dragging was used, a standard method for collecting questing ticks alongside flagging. Flagging is reportedly more efficient for adult ticks, particularly in spring and winter [65], which may explain the predominance of nymphs. While both methods can collect other species such as *D. marginatus*, *Hyalomma marginatum*, and *Haemaphysalis inermis*, these are absent in central Switzerland, consistent with our exclusive detection of *I. ricinus*.

Most ticks were collected from vegetation at forest–meadow edges, with fewer found along forest paths or in open meadows. *I. ricinus* thrives in humid, mild habitats with dense vegetation and abundant hosts. Ecotones such as forest edges provide ideal conditions, retaining moisture and attracting wildlife [66]. The lower tick abundance observed in meadows and along paths likely reflects reduced humidity and the absence of leaf litter needed to maintain stable microclimates.

The detection of *Cytauxzoon* spp. in questing ticks collected from the vegetation speaks against the possibility that the uptake of blood from an infected cat caused the PCR-positive signal, but suggests that *I. ricinus* could play a role in the transmission cycle of *Cytauxzoon* spp. The detection in nymphs is particularly noteworthy, as it supports the possibility of transstadial transmission (from larva to nymph) following a larval blood meal on a competent reservoir host. Transovarial transmission, i.e., the passage from an infected female tick to her offspring without prior host feeding, may also be possible, but further experimental studies would be required to explore and confirm this hypothesis. This transmission mode would have important implications for the persistence and spread of the pathogen in the *I. ricinus* population [67]. Transovarial transmission has been confirmed for *Babesia canis* in *D. reticulatus* ticks in Europe [68], but it has not yet been investigated in *Cytauxzoon* spp., including *C. felis* [69]. Although *Cytauxzoon* spp. PCR-positive nymphs were detected in 2019, the absence of positive ticks in follow-up surveys in the same area several years later raises doubts about the occurrence of transovarial transmission. However, it should be noted that the ticks in later years were collected near, but not at, the exact location where the PCR-positive nymphs were originally detected. Moreover, the extent of general tick infestation with *Cytauxzoon* spp. in 2019 in the area remains unknown, and a depletion of the transmission cycle in case of strict tick prevention in cats might have happened.

The *Cytauxzoon* spp. qPCR assay used to screen tick DNA for the presence of the hemoparasite demonstrated high sensitivity when applied to blood samples from felids [21,25]. Moreover, in silico, the assay showed high specificity for detecting *Cytauxzoon* spp., which was confirmed using blood samples from infected cats [25]. However, when applied to investigating ticks, this same assay yielded positive results for all tested samples. Therefore, each sample pool underwent additional verification using conventional PCR, targeting a segment of the 18S rRNA gene of *Cytauxzoon* spp. to confirm the presence of parasite-specific sequences in the tick pools, and many of them tested negative. Moreover, there was no correlation between the Ct value of the qPCR and the results in the conventional PCR. The qPCR Ct values from ticks collected in 2022 and 2024 were comparable to those from the samples collected in 2019. At the same time, some of the 2019 samples tested positive, but all of the 2022/2024 samples tested negative by conventional PCR. We therefore hypothesize that the qPCR lacks specificity when applied to tick DNA, as it co-amplifies DNA from tick endosymbionts. Ticks host a diverse array of endosymbiotic bacteria, which are crucial for their survival and reproduction, and may play a possible role in the transmission dynamics of tick-borne diseases [70,71]. Therefore, the potential for cross-amplification of endosymbiont DNA in qPCR assays targeting the *Cytauxzoon* spp. 18S rDNA gene necessitates careful interpretation and confirmation of results, particularly when analyzing tick-derived DNA.

## 5. Conclusions

Cytauxzoonosis is a significant vector-borne disease of felids, with potentially serious health consequences for infected animals. While substantial progress has been made in recent years regarding the presence and geographic distribution of various *Cytauxzoon* spp. in Europe as well as worldwide, information on the associated tick vectors remains limited. This study provides the first evidence that *I. ricinus* may play a role in the transmission cycle of European *Cytauxzoon* spp. We found that the 18S rRNA qPCR assay used in this study for screening tick samples demonstrated limited specificity. Consequently, sequencing of longer amplicons represents an essential confirmatory step to address this methodological limitation. Future studies should aim to achieve species-level identification in order to establish a definitive link between the *Cytauxzoon* spp. detected in ticks and those infecting felids from the same region.

The results of this study provide a foundation for future epidemiological studies aimed at confirming vector competence, clarifying transmission dynamics, and determining whether *I. ricinus* or other tick species in Europe play a role in the life cycle of *Cytauxzoon* spp.

## Figures and Tables

**Table 1:** PCR assays used for screening, confirmation, and sequencing in this study.

Assay	Gene *	Primer/Probe ID	Sequence (5′-3′) #	Amplicon Length (bp)	Reference
qPCR (screening)	18S rRNA	Cytsp. 1525f	GAA TGC CTA GTA GAC GCG AGT CA	96	[21]
		Cytsp. 1593r	ACG GGC GGT GTG TAC AAA G		
		Cytsp. 1549p	6FAM-CAG CTC GTG TCG ATT ACG TCC CTG C-TAMRA		
Confirmatory/sequencing PCR	18S rRNA	Cytfelis.203f	AGA CCY YAA ACC ATC CCG CT	221	[21]
		Cytfelis.423r	CCT GCT GCC TTC CTT AGA TG		
Sequencing PCR	18S rRNA	Cytlblynx.23f	GCC ATGCAT GTC TAA GTA TAA GC	1637	[21]
		Cytlblynx.1659r	CGC GCC TAA CGA ATTAGA AG		
Sequencing PCR	CytB	Cytaux_cytb_F1	CTT AAC CCA ACT CAC GTA CC	1434	[34]
		Cytaux_cytb_R3	GGT TAA TCT TTC CTA TTC CTT ACG		
		Cytaux_cytb_Finn	ACC TAC TAA ACC TTA TTC AAG CRT T	1333	
		Cytaux_cytb_Rinn	AGA CTC TTA GAT GYA AAC TTC CC		
Sequencing PCR	COI	Th-For2	TGGYTKGCTTATTGGTTTGG	1966	[34]
		Piro_mt_R1	ACTTTGAACACACTGCTCG		
		Th-For2	TGG YTK GCT TAT TGG TTT GG	1656	
		Cytaux_260R	AAT TCC CAT CTC GCT ATC ACT TTC		

* rRNA: ribosomal ribonucleic acid; CytB: cytochrome b; COI: cytochrome oxidase subunit I; #: 6FAM: 6-carboxyfluorescein reporter; TAMRA: Carboxytetramethylrhodamine quencher.

**Table 2:** Characteristics of the investigated *I. ricinus* tick pools collected in 2019 and *Cytauxzoon* spp. PCR results.

IDs of Pools	Origin ^1^	Stage ^2^	Observations	Ticks/ Pool (Total Number of Ticks)	*Cytauxzoon* spp. qPCR Results (Ct Values)	*Cytauxzoon* spp. Conventional PCR Result	Positive Pools and Length of Amplicons (bp)
1–6	2 cats (?)	F	Engorged and not engorged	2–3 (16)	All positive (23.5–26.5)	All negative	NA
7	2 cats (?)	M	Not engorged	3 (3)	Positive (26.5)	**Positive**	Pool No. 7: 1535
8	Vegetation	F	NA	2 (2)	Positive (24.8)	Negative	NA
9–27	Vegetation	N	NA	5–6 (96)	All positive (24.7–26.5)	Negative (n = 16) **Positive** **(n = 3)**	Pool No. 13: 219; Pool No. 17: 219; Pool No. 19: 140
28–31	1 cat (+)	F	Engorged and not engorged	3 (12)	All positive (22.4–24.0)	All negative	NA
32–34	1 cat (+)	M	Not engorged	3 (9)	All positive (24.7–26.4)	All negative	NA
35 *	1 cat (+)	N	NA	1 (1)	Positive (26.5)	Negative	NA
36–41	4 cats (−)	F	Not engorged	3 (18)	All positive (22.3–25.4)	All negative	NA
42	4 cats (−)	M	Not engorged	1 (1) ^ꝉ^	Positive (26.6)	Negative	NA
42 pools	22 pools from cats, 20 pools from vegetation	14 males 49 females 97 nymphs		160 ticks 62 ticks from cats 98 ticks from vegetation	42 pools positive	4 pools positive 38 pools negative	

^1^ (?) = cat with unknown *Cytauxzoon* spp. infection status; (+) = *Cytauxzoon europeaus* positive cat; (−) = *Cytauxzoon* spp. negative cat; ^2^ M = male; F = female; N = nymph. NA = not applicable; * Pool 35 contained only 1 tick; ^ꝉ^ allocation of the tick to the 4 cats in the same household was not possible.

**Table 3:** Characteristics of the investigated questing *I. ricinus* tick pools collected in 2022 and 2024 and *Cytauxzoon* spp. PCR results.

IDs of Pools or Single Ticks	Year	Origin	Stage ^1^	Ticks/ Pool (Total Number of Ticks)	*Cytauxzoon* spp. qPCR Results (Ct Values)	*Cytauxzoon* spp. Conventional PCR Result
1–7	2022	Forest edge	N	1 (7)	All positive (25.4–27.5)	All negative
8–19	2024	Forest edge	F	1–3 (20)	All positive (22.7–25.1)	All negative
20–30	2024	Forest edge	M	1–3 (22)	All positive (25.3–28.2)	All negative
31–114	2024	Forest edge	N	2–5 (374)	All positive (23.3–28.8)	All negative
115–120	2024	Forest path	F	1–3 (12)	All positive (21.9–23.9)	All negative
121–127	2024	Forest path	M	1–3 (14)	All positive (24.5–30.0)	All negative
128–134	2024	Forest path	N	2–5 (23)	All positive (22.9–28.7)	All negative
135–136	2024	Meadow	F	1 (2)	All positive (22.4–24.2)	All negative
137–143	2024	Meadow	N	4–5 (31)	All positive (24.4–26.0)	All negative
143 pools		423 ticks from the forest edge 49 ticks from the forest path 33 ticks from the meadow	36 males 34 females 435 nymphs	505 ticks	143 positive	143 negative

^1^ N = nymph; M = male; F = female.

## Data Availability

The original contributions presented in this study are included in the article. Further inquiries can be directed to the corresponding author.
